# Purple urine bag syndrome: An unusual presentation of urinary tract infection: A case series and literature review

**DOI:** 10.1097/MD.0000000000044638

**Published:** 2025-09-19

**Authors:** Mohammed F. Basehi, Fatimah H. Dallak, Atheer I. Darraj, Sultan J. Almalki

**Affiliations:** a Emergency Department, King Fahd Central Hospital, Jazan Health Cluster, Jazan, Saudi Arabia; b Internal Medicine Department, King Fahd Central Hospital, Jazan Health Cluster, Jazan, Saudi Arabia; c Internal Medicine Department, Prince Mohammed Bin Nasser Hospital, Jazan Health Cluster, Jazan, Saudi Arabia.

**Keywords:** *Candida tropicalis*, *Escherichia coli*, indwelling catheter, purple urine bag syndrome, urinary tract infection

## Abstract

**Rationale:**

Purple urine bag syndrome (PUBS) is a rare but visually alarming condition associated with urinary tract infections (UTIs), typically occurring in debilitated elderly patients with long-term indwelling catheters. Awareness of PUBS is essential, as it can serve as an indicator of underlying infection.

**Patient concerns:**

Two elderly male patients presented with striking purple discoloration of their urinary catheter bags. Caregivers expressed concern, although both patients denied fever, dysuria, or suprapubic pain.

**Diagnoses:**

PUBS secondary to UTI was diagnosed. Case 1 involved mixed bacterial growth, with risk factors including diabetes mellitus, benign prostatic hyperplasia, immobility, and prolonged catheterization. Case 2 had *Candida tropicalis* infection, with predisposing factors of stroke-related immobility, constipation, and chronic catheterization.

**Interventions:**

Both patients underwent a Foley catheter and urine bag replacement. Case 1 received empirical oral ciprofloxacin, while case 2 required admission, intravenous antifungal therapy, and supportive hydration.

**Outcomes:**

Purple discoloration resolved after catheter replacement. Both patients achieved favorable outcomes without recurrence.

**Lessons:**

While PUBS is typically benign, it reflects underlying UTIs and requires prompt intervention. Early recognition, catheter management, and appropriate antimicrobial therapy are key to preventing complications. Clinician awareness is vital to avoid misdiagnosis and unnecessary anxiety.

## 1. Introduction

Purple urine bag syndrome (PUBS) is distinguished by purple urine and/or staining of the collection bag. Urine can appear purple, orange, or brown in color.^[1]^ PUBS is an uncommon but alarming condition. It causes fear and anxiety in the patient, the family, and the unaware health worker. It was first documented in 1978 by Barlow et al in The Lancet,^[2]^ but it had reportedly affected King George III in 1812 during an episode of chronic constipation. However, it remains an unusual phenomenon.^[3]^

It is usually encountered in debilitated patients with a history of long-term indwelling urinary catheters, with a prevalence of 9.8% among institutionalized patients and up to 42.1% in nursing and care homes.^[4,5]^ PUBS is a rare and unusual manifestation of a urinary tract infection (UTI). It is mostly documented in older women with debilitating medical conditions such as cancer, stroke, or dementia, and a history of long-standing indwelling urinary catheters, with or without constipation.

The pathogenesis and mechanism of PUBS involve a sequence of reactions related to the dietary digestion and intestinal absorption of tryptophan. The diagnosis is visually apparent and is usually straightforward with the change or removal of a Foley catheter, urine examination and culture, administration of appropriate antibiotics, and use of laxatives if constipation is present.^[1,6–8]^

Even though PUBS is typically thought to be benign, it is important to evaluate each patient to rule out an underlying UTI, especially in those who might have trouble communicating their symptoms, such as very ill or neurologically impaired patients.^[6,9]^ The lack of awareness in both patients and healthcare providers is a major factor that may lead to mistreatment and must be avoided for a better outcome. Gaining an understanding of PUBS is crucial for improving patient care.^[3,6]^

## 2. Methods

This study presents 2 cases of PUBS in elderly male patients, aiming to describe their clinical characteristics, contributing factors, and management approaches. To contextualize these findings, a comprehensive narrative review of the literature was conducted, focusing primarily on studies indexed in PubMed and supplemented by searches in Scopus, Web of Science, and Google Scholar. The review covered PUBS cases reported between 1993 and 2024, with the goal of identifying common etiological mechanisms, frequently isolated pathogens, and treatment strategies. As this was a nonsystematic review, selection bias and incomplete coverage of the literature cannot be excluded. Therefore, the findings should be interpreted as descriptive rather than exhaustive.

## 3. Case presentation

### 3.1. Case 1

A 68-year-old Saudi male, with a background history of type 2 diabetes mellitus on oral hypoglycemic agents and right-sided hemiplegia secondary to stroke for a duration of 3 years, was on medications and physiotherapy. The patient had a bladder outlet obstruction due to benign prostatic hyperplasia and was on a urethral (Foley) catheter. He had a history of multiple failed trials of voiding without the catheter. He had been using a Foley urethral catheter for 3 years, with regular changes every 4 weeks.

**Figure 1.**
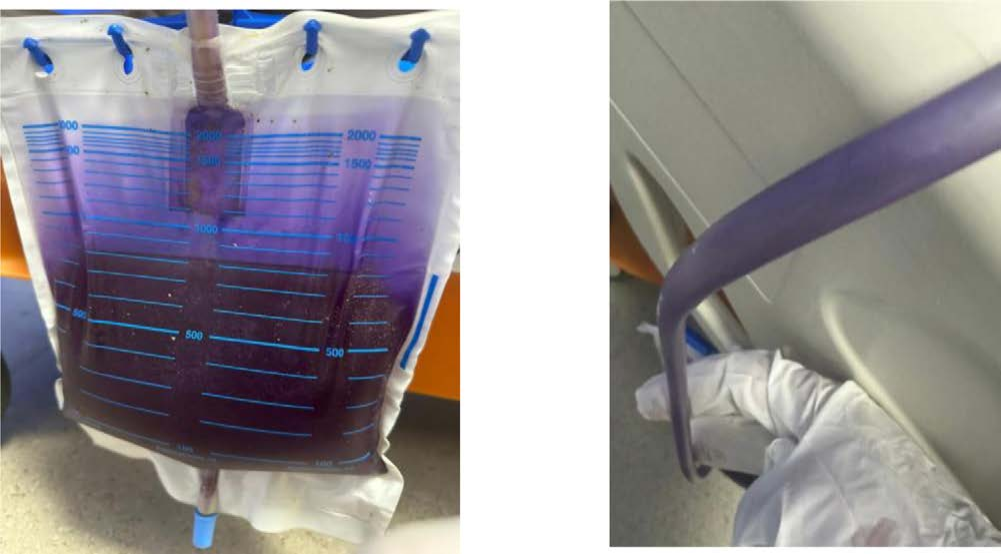
Purple urine is visible in both the tubing and urine collection bag of Case 1, demonstrating the classical discoloration associated with PUBS. PUBS = purple urine bag syndrome.

The patient was brought by his family to the emergency department with a complaint of purple discoloration of the urine collection bag (Fig. 1A and B), associated with foul-smelling urine for 2 days and poor oral intake. He denied any fever, dysuria, pyuria, backache, constipation, or suprapubic pain, and denied any history of weight loss. There was no history of drug or herb consumption that could potentially have produced purple urine. His activity had been largely restricted to bed in the last few years.

The vital signs were stable and afebrile. Physical examination revealed a conscious, oriented patient with a soft and lax abdomen and a purple urine bag. Laboratory tests showed: white blood cell count (WBC) 5.9 × 10⁹/L, hemoglobin 14 g/dL, platelet count 250 × 10⁹/L, serum creatinine 107 µmol/L, and serum urea 12.2 mmol/L.

Urine dipstick showed nitrate (+), blood (4+), and protein (1+). Urinalysis showed leucocyte esterase (2+), leucocytes 0 to 10/HPF, erythrocytes 0 to 2/HPF, bacteriuria (3+), blood (2+), and nitrate (1+).

The Foley catheter was changed, and the purple color disappeared after changing the catheter and the urine bag. Urine culture showed mixed growth. As the patient was asymptomatic apart from the unexpected urine color noted by caregivers, he was treated with oral antibiotics as an outpatient with close follow-up by the home care team. He was given ciprofloxacin 500 mg twice daily empirically. He was followed up for 2 weeks, during which no further symptoms were reported, and the urine color remained clear.

### 3.2. Case 2

An 87-year-old Saudi male, with a background history of chronic hypertension on amlodipine 5 mg orally once daily and an old cerebrovascular accident 8 years ago, was on medications and physiotherapy. The patient was on a Foley catheter due to bedridden status as a result of a stroke and residual weakness. He had multiple hospitalizations for burning micturition and aspiration pneumonia.

**Figure 2.**
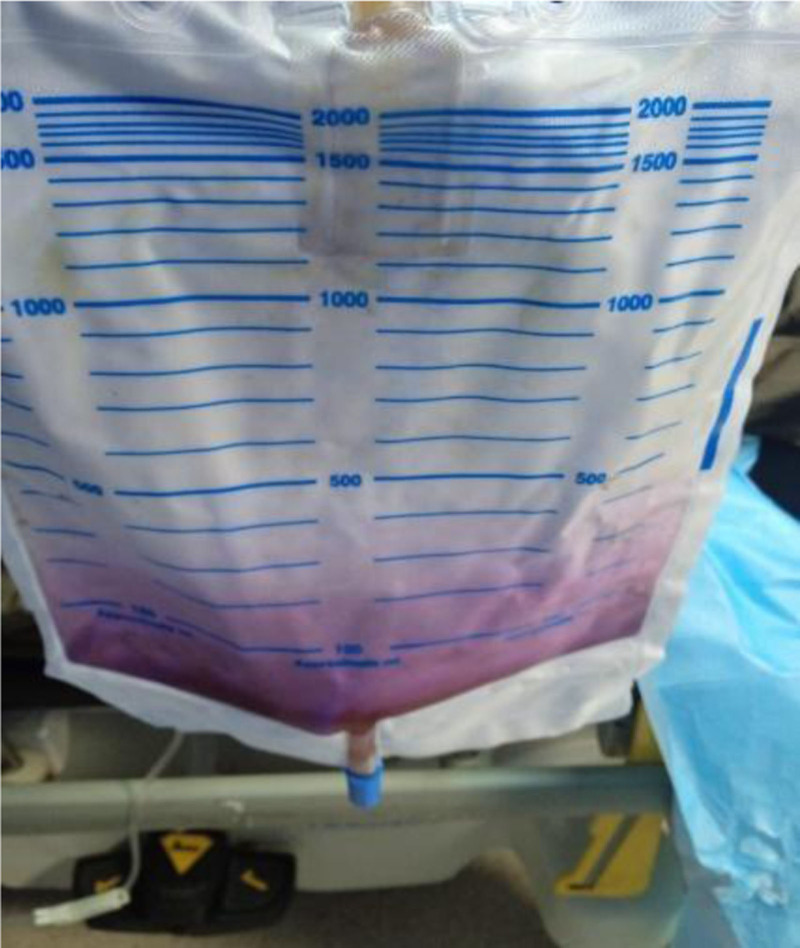
Purple discoloration of the urine collection bag in Case 2, highlighting the striking appearance of PUBS. PUBS = purple urine bag syndrome.

The patient was brought by his family to the emergency department with purple urine collected in a urine bag (Fig. 2) and poor oral intake associated with constipation for 3 days. He denied fever, dysuria, pyuria, backache, suprapubic pain, or any history of weight loss. There was no history of drug or herb consumption that could have potentially caused purple urine. His activity had been largely restricted to bed in the last few years.

The vital signs were stable and afebrile. Physical examination revealed a conscious, oriented patient with a soft and lax abdomen and a purple urine bag. Laboratory tests showed: WBC 7.1 × 10³/µL, hemoglobin 13.7 g/dL, creatinine 49.84 µmol/L, and urea 4.82 mmol/L. Urinalysis showed: RBCs+ per HPF, yeast 4+, WBCs (−), nitrate (−), and leukocyte esterase (−).

The patient was admitted for management of dehydration. The Foley catheter and urine bag were changed. Intravenous antibiotics were administered along with adequate hydration. Urine culture was positive for *C tropicalis*.

## 4. Literature review

Table 1 presents the purple urine bag syndrome clinical data review of identified cases reported in the literature. The reviewed cases highlight that PUBS predominantly occurs in elderly, debilitated patients. The mean age of affected individuals ranges from the late 60s to early 80s, with reported cases in patients as young as 16 and as old as 101. Gender distribution shows a slight predominance in females, particularly those with comorbidities such as vascular dementia, hypertension, or diabetes mellitus.^[10–98]^

**Table 1:** Purple urine bag syndrome clinical data review.

References	Gender	Age	Comorbidities	Signs, symptoms	Bacteria identified
Matsuo et al^[10]^	M	76	CVA with subsequent hemiplegia	Constipation	*Escherichia coli, Morganella morganii, Proteus mirabilis, Staphylococcus aureus, Enterococcus faecalis*
Matsuo et al^[10]^	M	86	DM, chronic kidney disease (CKD)	Constipation	*Escherichia coli, Morganella morganii, Proteus mirabilis, Providencia rettgeri*
Matsuo et al^[10]^	M	84	CVA with subsequent hemiplegia	None	*Escherichia coli, Morganella morganii, Proteus mirabilis, Enterococcus faecalis, Streptococcus agalactiae*
Al-Jubouri^[11]^	F	85	None	None	*Providencia rettgeri*
Ollapallil et al^[12]^	F	61	End stage renal disease (ESRD), DM	Constipation	Mixed Bacilli
Ollapallil et al^[12]^	F	49	Schizophrenia	None	NA
Ihama and Hokama^[13]^	F	93	Advanced gastric cancer	Epigastric pain	*Providencia stuartii, Alcaligene*
Fain-Ghironi et al^[14]^	F	85	None	None	*Providencia stuartii, Citrobacter koseri*
Ribeiro et al^[15]^	F	56	Amyotrophic lateral sclerosis	None	*Morganella morganii, Pseudomonas aeruginosa, Proteus mirabilis*
Vallejo-Manzur et al^[16]^	M	72	CKD, CVA, HTN, Parkinson disease	Lethargic	*Escherichia coli*
Wang et al^[17]^	M	61	ESRD, DM	Constipation	*Klebsiella pneumoniae*
Achtergael et al^[18]^	M	77	Prostate cancer, and urinary retention	None	*Aerobic mixed bacterial culture*
Tang^[19]^	M	70	Tuberculous meningitis, arachnoiditis	Constipation	*Proteus mirabilis*
Tang^[19]^	F	81	Massive intracranial hemorrhage	None	*Enterococcus*
Beunk et al^[20]^	F	93	Pelvic fracture	None	None listed
Gautam et al^[21]^	M	70	Acute retention for transurethral resection of the prostate (TURP)	None	*Escherichia coli*
Ting et al^[22]^	F	72	ESRD, DM, CVA	None	*Escherichia coli*
Lazimy et al^[23]^	F	74	Cervical cancer, uretero-colic fistula	None	*Gram negative polymophs*
Harun et al^[24]^	F	45	Squamous cell carcinoma (SCC) of the cervix	None	Contamination
Harun et al^[24]^	F	75	CVA	None	*Escherichia coli*
Pillai et al^[25]^	F	76	Parkinson disease, HTN, asthma, depression, CVA	Decrease oral intake, constipation	Mixed growth
Lin et al^[26]^	M	72	DM, ESRD	NA	NA
Lin et al^[26]^	M	72	DM, ESRD, Alzheimer dementia	NA	NA`
Lin et al^[26]^	M	83	DM, HTN, Alzheimer dementia	NA	*Escherichia coli, Proteus mirabilis*
Lin et al^[26]^	M	89	DM, ESRD, BPH, HTN	NA	*Escherichia coli, Proteus mirabilis*
Lin et al^[26]^	M	80	VD, HTN	Constipation	*Providencia rettgeri*
Lin et al^[26]^	F	80	Anemia, pulmonary fibrosis, hepatitis, hypercholesterolemia	NA	*Escherichia coli*
Lin et al^[26]^	F	76	VD, anemia	Constipation	*Klebsiella pneumoniae*
Lin et al^[26]^	F	66	VD, HTN, hepatitis, hypercholesterolemia	NA	*Klebsiella pneumoniae*
Lin et al^[26]^	F	60	Schizophrenia, VD, poliomyelitis	NA	*Providencia rettgeri*
Lin et al^[26]^	F	75	DM, VD, HTN, hypercholesterolemia	Constipation	*Klebsiella pneumoniae*
Tan et al^[27]^	M	58	NA	NA	*Proteus mirabilis*
Chung et al^[28]^	M	85	CKD, CVA, HTN	Constipation	*Pseudomonas aeruginosa, Enterobacter cloacae*
Muneoka et al^[29]^	M	100	Alzheimer-type dementia	NA	*Morganella morganii, Pseudomonas aeruginosa*
Muneoka et al^[29]^	M	86	Alzheimer-type dementia	NA	*Morganella morganii*
Muneoka et al^[29]^	F	93	Alzheimer-type dementia	NA	*Citrobacter, Escherichia coli*
Muneoka et al^[29]^	F	86	Alzheimer-type dementia	NA	*Morganella morganii*
Muneoka et al^[29]^	F	84	Alzheimer-type dementia	NA	*Klebsiella pneumoniae*
Muneoka et al^[29]^	M	77	Alzheimer-type dementia	NA	*Enterococcus faecalis*
Khellaf et al^[30]^	F	94	CVA	NA	*Proteus mirabilis*
Al-Sardar and Haroon^[6]^	M	82	HTN, depression, CVA	Abdominal pain, distention, and constipation.	Mixed
Wu et al^[31]^	F	95	Dementia, CKD,	NA	*Escherichia coli, Klebsiella pneumoniae, Proteus mirabilis*
Pillai et al^[32]^	F	68	DM	Fever	*Escherichia coli*
Ferrara et al^[33]^	F	81	DM with nephrotic range nephropathy, HTN, dyslipidaemia	NA	*Escherichia coli*
Siu and Watanabe^[34]^	M	48	DM, acute coronary syndrome (ACS), and history of coronary artery bypass.	NA	*Escherichia coli*
Su et al^[35]^	F	81	Bedridden status	Fever	*Proteus mirabilis*
Keenan and Thompson^[36]^	M	97	NA	Constipation	*Klebsiella pneumoniae*
Khan et al^[37]^	M	39	Cervical spine injury post road traffic accident (RTA) with subsequent paraplegia	Fever, chills, and sweating	*Providencia rettgeri, Pseudomonas auruginosa, Proteus mirabilis, Enterococcus faecalis.*
Peters et al^[38]^	F	82	NA	NA	*Proteus mirabilis*
Lee and Tan^[39]^	M	75	ESRD, DM, HTN, CVA, and degenerative spine disease	NA	*Proteus vulgaris*
Bocrie et al^[40]^	F	87	Post-fall syndrome	Urine retention	*Escherichia coli*
Cantaloube et al^[41]^	F	81	CVA, loss of autonomy, post cardiac arrest	NA	*Pseudomonas aeruginosa*
Meekins et al^[42]^	F	67	Congestive Heart Failure (CHF), transverse myelitis	Constipation	*Morganella morganii, Proteus mirabilis*
Montasir and Mustaque^[4]^	F	86	Osteoporosis, bilateral fracture of the femur neck for 2 years.	Constipation	*Escherichia coli*
Bhattarai et al^[43]^	F	87	ESRD, dementia, HTN, hyperlipidemia, recurrent UTI	Constipation	*Enterococci species, Pseudomonas aeruginosa*
Canavese et al^[44]^	F	60	CVA	NA	NA
Canavese et al^[44]^	M	78	ACS, HTN, and hypercholesterolemia	Oliguric	NA
Canavese et al^[44]^	M	89	BPH, cerebral vasculopathy, and laryngeal carcinoma	NA	*Providencia rettgeri*
Canavese et al^[44]^	F	99	Severe decline in her neurological status, CHF	NA	*Providencia stuartii, Enterococcus faecalis, Proteus mirabilis*
Su^[45]^	F	67	NA	Fever, malaise, vomiting, and GI upset	*Escherichia coli*
Su^[45]^	F	61	NA	NA	NA
Duff^[46]^	F	57	Transverse myelitis	3-day history of diffuse abdominal pain	*Klebsiella pneumonia*
Barreira et al^[47]^	M	92	BPH, arterial fibrillation, renal failure	NA	*Escherichia coli. Morganella morganii*
Barreira et al^[47]^	M	95	HTN, bladder neoplasia, ischemic heart diseases (IHD), and renal failure	NA	*Proteus mirabilis, Escherichia coli*
Mohamad and Chong^[48]^	F	78	Hyperlipidemia, HTN, and dementia	NA	*Proteus mirabilis*
Ungprasert et al^[49]^	M	44	Down syndrome, neurogenic bladder	Fever	*Enterococcus faecalis*
Wolff et al^[50]^	NA	90	Non-Hodgkin lymphoma	Urine retention	*Escherichia coli*
Yaqub et al^[51]^	F	83	Dementia	Nausea, vomiting, decreased oral intake, chronic constipation	*Escherichia coli*
Agapakis et al^[52]^	F	83	Hypothyroidism, Alzheimer disease, and colon cancer	Fever	*Escherichia coli*
Chassin-Trubert^[53]^	M	72	CKD, ACS, and obstructive uropathy	None	*Citrobacter freundii*
Delgado et al^[54]^	F	60	ESRD, hypothyroidism, DM, and HTN	NA	*Klebsiella pneumoniae*
Restuccia and Blasi^[55]^	F	81	Gastric cancer, CHF, chronic respiratory failure, HTN, DM, and multiple vertebral collapses.	NA	*Escherichia coli*
Abubacker et al^[1]^	M	36	Urinary incontinence following a fall from a tree	Constipation	*Providencia stuartii*
Alex et al^[8]^	M	83	BPH, CKD	None	*Klebsiella pneumonia, Morganella morgaii, Enterococcus, Citrobaterdiversus, Pseudomonas aeruginosa*
Karim et al^[56]^	M	83	Mild Alzheimer dementia, myelodysplastic syndrome, and bladder cancer	None	*Pseudomonas aeruginosa*
Kenzaka^[57]^	F	72	Bladder Cancer	None	*Escherichia coli*
Mohamed Faisal^[58]^	M	68	Paraplegia following RTA	None	Mixed growth
Mondragón^[59]^	F	71	HTN, CVA	None	*Escherichia coli, Proteus mirabilis, Enterococcus faecalis*
Redwood et al^[60]^	M	90	BPH	Suprapubic pain decreased oral intake.	*Escherichia coli*
Van Keer et al^[61]^	M	80	CKD, CHF, IHD, prostate cancer, chronic obstructive pulmonary disease (COPD), and DM	Burning sensation in the lower abdomen	*Escherichia coli.*
Van Keer et al^[61]^	M	81	left kidney atrophy, IHD, non-Hodgkin lymphoma	None	*Pseudomonas aeruginosa, Enterococcus faecalis*
Demelo-Rodríguez et al^[62]^	M	83	HTN, DM, COPD, and BPH	None	*Klebsiella pneumoniae*
Faridi et al^[63]^	M	76	BPH	Dysuria	*Escherichia coli*
Richardson-May^[64]^	F	94	CVA	None	*Gram-negative coliforms*
Sriramnaveen et al^[65]^	M	85	HTN, obstructive uropathy	Uremic symptoms	*Pseudomonas aeruginosa*
Llah et al^[66]^	M	52	Quadriplegia secondary to trauma, depression	Right lower abdominal pain, Fever, chills, and nausea	*Morganella species*
Traynor et al^[67]^	F	90	VD, femur fracture, thromboembolic disease, and osteoporosis.	None	Mixed growth
Sulaiman et al^[68]^	F	65	CVA	None	*Klebsiella pneumonia*
Golzarri^[69]^	M	57	Non-Hodgkin lymphoma, paraplegic following spinal cord compression	Fever	*Klebsiella pneumonia*
Guei et al^[70]^	F	47	ESRD, and HTN	Headache and dizziness.	*Escherichia coli*
Wong and Abdullah^[71]^	F	86	Urinary retention	None	Mixed growth
Karray et al^[72]^	M	78	HTN, DM, prostatic adenocarcinoma	Fever	*Escherichia Coli*
Shin et al^[73]^	F	81	HTN, and CHF	Loss of appetite, and nausea	*NA*
Shin et al^[73]^	F	88	HTN	None	*NA*
Kumar et al^[74]^	F	56	Breast cancer	None	*NA*
Kumar et al^[74]^	F	75	Non-Hodgkin lymphoma, and neck femur fracture	None	*NA*
Worku^[75]^	F	94	CVA and chronic constipation	Constipation	*Escherichia coli*
Vikse et al^[76]^	M	70	Myelodysplastic syndrome, CKD, and BPH	None	*Proteus vulgaris, Enterococcus faecalis*
Wattanapisit et al^[77]^	F	89	CVA, HTN	None	NA
Wattanapisit et al^[77]^	F	70	Dementia, DM, HTN, and dyslipidemia	None	NA
Wattanapisit et al^[77]^	F	88	Lymphoma	None	NA
Wattanapisit et al^[77]^	F	91	Colon cancer, and liver cirrhosis	None	*Escherichia coli*
Ong and Vasanwala^[78]^	M	50	DM, and BPH	None	NA
De Menezes Neves et al^[79]^	M	84	HTN, Parkinson disease, prostate cancer with bladder invasion	Lower back pain	*Proteus penneri, Enterococcus faecalis*
Kumar et al^[80]^	M	60	Paraplegia and urinary incontinence following spinal cord injury	None	*Escherichia coli*
Hien et al^[81]^	F	16	ESRD, seizure, and HTN	None	*Escherichia coli*
Hien et al ^[81]^	F	30	ESRD	Uremic symptoms	NA
Gago et al^[7]^	F	93	Rectal adenocarcinoma	Fever	*Escherichia coli*
Kumar et al^[82]^	F	52	Neurogenic bladder following trauma, chronic constipation	Nausea, fever	*Escherichia coli*
Saraireh et al^[83]^	F	80	DM, ESRD, HTN, IHD	Constipation	*Proteus mirabilis*
Li and Sud^[84]^	F	77	ESRD	None	NA
Popoola and Hillier^[85]^	F	90	Acute decompensated heart failure (ADHF)	None	*Proteus species*
DeRon and Legan^[86]^	F	79	HTN, CVA and neurogenic bladder, dementia	Confusion	*Proteus mirabilis, Escherichia coli*
Kannan and Bauman^[87]^	F	76	CKD, CHF, bladder cancer	SOB, constipation	*Proteus mirabilis*
Tirtayasa et al^[88]^	F	27	Neurogenic bladder and paraparesis	None	*Escherichia coli, Pseudomonas aeruginosa, Klebsiella pneumonia, gram-negative bacteria*
Jappi and Hadi^[89]^	F	23	Lupus nephritis	None	*Enterococcus faecalis*
Vanumu et al^[90]^	F	73	Vaginal cancer	Fever, constipation, SOB	*Escherichia coli, Klebsiella pneumonia*
Popović et al^[91]^	F	79	HTN, spinal compression fracture and neurogenic bladder	None	*Proteus mirabilis*
Shaik et al^[92]^	M	64	Trisomy 21, hypothyroidism, adrenal insufficiency, seizure, chronic respiratory failure	None	NA
Faia et al^[93]^	F	87	Parkinson disease, CVA, dyslipidemia, HTN, hypothyroidism, urinary retention	Confusion	*Providencia stuartii*
Julião and Cruz^[94]^	F	98	Alzheimer dementia, HTN, hypercholesterolemia, chronic constipation	None	*Escherichia coli*
Pereira et al^[95]^	F	81	Urothelial carcinoma	None	*Morganella morganii*
Wang et al^[96]^	F	101	Chronic constipation, intertrochanteric femur fracture	None	NA
Murray et al^[97]^	F	46	Cerebral palsy, breast cancer, deep vein thrombosis (DVT)	None	*Escherichia coli*
Mahdi and Larijani^[98]^	F	73	CVA, DVT	Fever, uremic symptoms	*Klebsiella pneumonia*

BPH = benign prostatic hyperplasia, DM = diabetes mellitus, NA = no data available, SOB = shortness of breath.

### 4.1. Demographic analysis

**Figure 3.**
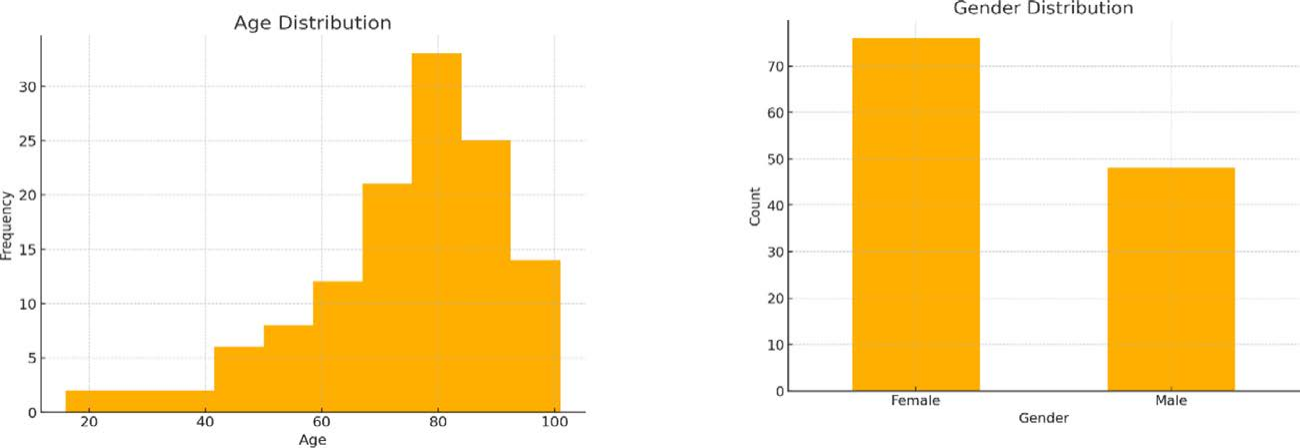
Distribution of PUBS cases by age and gender based on the reviewed literature, showing predominance among elderly females. PUBS = purple urine bag syndrome.

Figure 3 presents the distribution of PUBS cases by age and gender based on the reviewed literature, showing predominance among elderly females. The dataset includes both male and female patients, with females accounting for 61.29% of cases and males 38.71%. Most cases are concentrated in older age groups, with a mean age of 74.34 years. The minimum recorded age is 16 years, while the maximum is 101 years. The data also reveal a median age of 78 years, with the 25th percentile at 66 years and the 75th percentile at 86 years.

The most commonly reported symptom was either an absence of symptoms or unspecified records, observed in 76 cases. Constipation was a frequently noted symptom, reported in 21 cases. Fever was recorded in 13 cases with consistent presentation. Other symptoms included abdominal pain, which was recorded in 4 cases, nausea observed in 3 cases, vomiting, and shortness of breath, each reported in 2 cases.^[10–98]^

The dataset also summarizes the distribution of bacterial species identified in reported cases, as shown in Table 2. Bacterial species identified in reported PUBS cases. The most frequently encountered organism was *Escherichia coli*, observed in 42 cases, followed by *Proteus mirabilis* in 21 cases and *Klebsiella pneumoniae* in 16 cases. Additionally, 9 entries were unspecified or left blank, and testing was not performed in 18 cases. Other notable organisms included *Morganella morganii* (11 cases), *Pseudomonas aeruginosa* (11 cases), and *Enterococcus faecalis* (11 cases). Less common pathogens, such as *Providencia rettgeri* and *Providencia stuartii*, were each reported in 5 cases.^[10–98]^

**Table 2:** Bacterial species identified in reported cases.

Bacterial species	Number of cases
*Escherichia coli*	42
*Proteus mirabilis*	21
*Klebsiella pneumoniae*	16
*Empty or unspecified entries*	9
*Not Performed*	18
*Morganella morganii*	11
*Pseudomonas aeruginosa*	11
*Enterococcus faecalis*	11
*Providencia rettgeri*	5
*Providencia stuartii*	5

## 5. Discussion

PUBS is an intriguing yet rare clinical condition characterized by the striking purple discoloration of urine collection bags. Although visually alarming, PUBS is typically benign but may indicate an underlying UTI that requires medical attention. The syndrome is more frequently observed in elderly, debilitated patients with chronic conditions requiring long-term catheterization. This report discusses 2 cases, highlighting their clinical presentations, underlying pathophysiology, and management.^[1]^

### 5.1. Clinical presentations and predisposing factors

Both cases involved elderly males with chronic illnesses and long-term indwelling Foley catheters (classic risk factors for PUBS). The first patient, a 68-year-old male, had a history of diabetes, stroke, and benign prostatic hyperplasia. The second, an 87-year-old male, was bedridden following a cerebrovascular accident and experienced recurrent infections. These cases underscore the vulnerability of such patients to PUBS due to:

1. *Catheter use*: Indwelling catheters increase the risk of biofilm formation, which promotes bacterial colonization and metabolic activity, leading to pigment production.^[6]^
2. *Comorbidities*: Chronic illnesses like diabetes and stroke compromise immune defenses, creating a favorable environment for infections.^[1,7]^
3. *Immobility*: Both patients were bedridden, reducing physical activity and contributing to complications like constipation, which is also associated with PUBS.^[3]^

### 5.2. Pathophysiology of PUBS

The pathogenesis involves the metabolic breakdown of dietary tryptophan into indole, which is absorbed in the gut, metabolized to indican in the liver, and excreted in the urine. In the presence of bacteria producing enzymes such as tryptophanase, indican is further degraded to indigo (a blue pigment) and indirubin (a red pigment), leading to the purple discoloration of the urine.^[1]^ This condition is facilitated by alkaline urine, bacterial biofilms, and prolonged catheter use.^[5]^

### 5.3. Diagnostic and laboratory findings

In both cases, the diagnosis was visually apparent and supported by laboratory findings. Urine dipstick analysis in the first case revealed leukocyte esterase, nitrates, and bacteriuria, while culture results showed mixed bacterial growth. The second case involved *C tropicalis* as the causative organism, highlighting the potential role of fungi in PUBS. These findings emphasize the importance of culture testing to guide antimicrobial therapy.

### 5.4. Management and outcomes

Management of PUBS primarily involves addressing the underlying infection, replacing the contaminated catheter, and alleviating predisposing factors such as constipation. In the first case, oral ciprofloxacin and catheter replacement led to complete resolution. The second case required intravenous antifungal therapy due to *Candida* infection. Both patients showed favorable outcomes with no recurrence of PUBS. While PUBS is most frequently associated with *E coli* and *P mirabilis*, empirical therapy should be guided by local antibiogram data and antimicrobial stewardship principles to ensure efficacy and minimize resistance.^[3,99]^

## 6. Conclusion

This study highlights the clinical significance of purple urine bag syndrome as a rare, visually striking manifestation of UTIs. PUBS serves as an important diagnostic marker, particularly in debilitated patients with chronic illnesses and long-term catheterization. While PUBS is a benign condition, it often indicates underlying UTIs that require prompt diagnosis and management. Awareness among healthcare providers is essential to ensure timely identification, appropriate treatment, and prevention of complications. The findings reinforce the need for targeted interventions such as prompt catheter replacement, correction of predisposing factors like constipation, and administration of appropriate antimicrobial therapy based on culture results. Continued research and reporting of PUBS cases can further enhance understanding and improve patient outcomes.

## Acknowledgments

The authors would like to thank the patients and their families.

## Author contributions

**Conceptualization:** Mohammed F. Basehi, Fatimah H. Dallak, Atheer I. Darraj.

**Data curation:** Mohammed F. Basehi, Atheer I. Darraj.

**Formal analysis:** Mohammed F. Basehi.

**Investigation:** Mohammed F. Basehi.

**Methodology:** Mohammed F. Basehi, Atheer I. Darraj, Sultan J. Almalki.

**Project administration:** Mohammed F. Basehi.

**Resources:** Mohammed F. Basehi, Fatimah H. Dallak.

**Software:** Mohammed F. Basehi.

**Supervision:** Fatimah H. Dallak.

**Validation:** Mohammed F. Basehi, Fatimah H. Dallak.

**Visualization:** Mohammed F. Basehi, Fatimah H. Dallak.

**Writing – original draft:** Mohammed F. Basehi, Fatimah H. Dallak, Atheer I. Darraj, Sultan J. Almalki.

**Writing – review & editing:** Fatimah H. Dallak, Sultan J. Almalki.
